# Superficial Mycoses Associated with Diaper Dermatitis

**DOI:** 10.1007/s11046-016-0020-9

**Published:** 2016-05-19

**Authors:** Alexandro Bonifaz, Rubí Rojas, Andrés Tirado-Sánchez, Dinora Chávez-López, Carlos Mena, Luz Calderón, Ponce-Olivera Rosa María

**Affiliations:** 1 Department of Mycology and Dermatology Service, Hospital General de México, “Dr. Eduardo Liceaga”, Dr. Balmis 148, Col Doctores, CP 06720 Mexico, DF Mexico; 2 Dermatology Service, Hospital Infantil de México, “Dr. Federico Gómez”, Mexico, Mexico

**Keywords:** Diaper dermatitis, *Candida albicans*, Dermatophytosis, *Epidermophyton floccosum*, *Malassezia* spp.

## Abstract

Diapers create particular conditions of moisture and friction, and with urine and feces come increased pH and irritating enzymes (lipases and proteases). Fungi can take advantage of all these factors. *Candida* yeasts, especially *C. albicans*, are responsible for the most frequent secondary infections and are isolated in more than 80 % of cases. Correct diagnosis is important for ensuring the correct prescription of topical antimycotics. Nystatin, imidazoles and ciclopirox are effective. It is important to realize there are resistant strains. Dermatophytes can infect the diaper area, with the most common agent being *Epidermophyton floccosum*. The clinical characteristics of dermatophytosis are different from those of candidiasis, and it can be diagnosed and treated simply. *Malassezia* yeasts can aggravate conditions affecting the diaper area, such as seborrheic dermatitis, atopic dermatitis, and inverse psoriasis. Additional treatment is recommended in this case, because they usually involve complement activation and increased specific IgE levels. Erythrasma is a pseudomycosis that is indistinguishable from candidiasis and may also occur in large skin folds. It is treated with topical antibacterial products and some antimycotics.

## Introduction

Disposable diapers were first produced in the 1940s, but were initially considered to be luxury items. It was not until the 1960s that they began to be used on a mass scale. By then, diapers were made with layers of cellulose, which made them more absorbent and resistant. [1–3]. However, they can also cause diaper dermatitis (DD), also known as diaper rash, which can be associated with different infections, especially *Candida* infections [3]. Other superficial mycotic conditions commonly found in the diaper area are dermatophytosis or tinea infections, exacerbation of seborrheic dermatitis by *Malassezia* yeasts and some pseudomycotic conditions like erythrasma [4–6]. Since diapers are mainly used for babies, most complications occur among this age group, but it must be remembered that some elderly people also use diapers for some reasons.

Our main objective in this study is to review superficial mycoses of the diaper area and their clinical, epidemiological, and diagnostic characteristics and treatment.

## Candidiasis Associated with Diaper Dermatitis

### Epidemiological, Pathogenic and Clinical Forms

DD is a common condition, especially among newborns and infants. It is an irritating and inflammatory acute dermatitis in the perineal and perianal areas resulting from occlusion and irritation caused by diapers. Most cases clear up in a day without treatment, but cases that last 3 days or more are more complicated and may be associated with infections [1–3]. Prevalence and incidence are high, varying under the conditions of each country and the most commonly used diaper. DD prevalence is estimated at 7–35 %, and incidence is highest in infants between 9 and 12 months of age [3, 6–9]. In a recent study conducted in the UK [10], for example, the prevalence of diaper rash was 25 % in the first month after birth, contrasting with the first studies carried out in the 1980s, which revealed a prevalence of almost 70 % [1–3, 6, 8, 11].

The first studies inferred the main triggering factor was increased pH caused by microflora [3, 6, 8, 9]. Although this is normally an important factor, we now know that DD development is much more complex and multifactorial, involving a series of orchestrated processes [12–22], all of these are summarized in Table 1.

**Table 1:** Main predisposing factors for DD and their mechanisms of action

Predisposing factor	Mechanism of action
Moisture [1, 3, 12]	Increased relative moisture of the skin Damage to the skin barrier
Friction [3, 10, 11]	Friction between diaper and damp skin increases skin damage (convex regions)
Urine [1, 3, 9]	Presence of urea causes irritation and is the base product for conversion to ammonia
Feces [1, 3, 9, 13, 20]	The presence of lipases and proteases causes skin damage with filaggrin proteolysis and increased NMF^a^ and TWEL^b^
Ammonia and increased pH [1, 3, 12, 13]	Conversion of urea to ammonium hydroxide by bacterial flora (main responsible, *Bacillus ammoniagenes)* Increase in pH from 5.5 to 6.8–7.15
Microorganisms [1, 3, 12, 13, 16–19, 21, 22]	Bacteria *Staphylococcus aureus* (isolated most frequently) Other: (β-hemolytic) *Streptococcus* sp.*, E. coli* and *Bacteroides* sp. More than 80 % *C. albicans* (80−90 %) Other: *C. tropicalis, C. parapsilosis, C. glabrata*
Use of antibiotics [1, 3, 9, 21, 22]	Use of broad-spectrum antibiotics causes increased *Candida* spp.
Association with other conditions [1, 3, 11]	Seborrheic dermatitis, inverse psoriasis, epidermolysis bullosa, granuloma gluteale infantum and acrodermatitis enteropathica

^a^ *NMF* natural moisturizing factor

^b^ *TEWL* transepidermal water loss

The most common infections associated with DD are caused by *Candida* yeasts, especially *Candida albicans*, which has been reported in more than 80 % [3, 6, 7, 17, 18]. These yeasts also cause secondary infections. Normally the number of *Candida* yeasts in the diaper area without dermatitis is low and such yeasts have been isolated in <4 % of cases [18, 19], while they are present between 70 and 92 % of children with DD [8, 9, 20]. The origin of these yeasts is directly related to the intestinal flora, and they have been isolated in children with oral and esophageal candidiasis (thrush). The clinical symptoms in these cases are more severe because the yeasts are excreted in the feces. [7–9, 21, 22].

A series of factors favor *Candida* infection in the diaper area. They are mostly acidophilic yeasts that thrive at skin pH, which is around 5.5, or 6.0 in newborns (owing to vernix caseosa and amniotic fluid), tending to normalize in a few days [1, 3, 17, 18, 22]. *C. albicans* and other yeasts provide examples of perfect adaptation to pH changes, which is controlled by two genes: *PHR2*, which is activated in acidic environments and is deactivated when pH increases, and *PHR1*, which does the opposite, i.e. is activated at a high pH (neutral and basic levels) [23]. Another proven factor in developing fungi (yeasts and dermatophytes) is CO_2_ levels, which are higher in the occlusive environment of standard disposable diapers and barely detectable in breathable diapers [16, 24, 25].

*Candida albicans*, settle infection, causing increased yeast numbers and a micromorphological change from blastoconidia to hyphae and pseudohyphae, which penetrate the superficial parts of the stratum corneum and epidermis [18, 19, 25]. In general, secondary *Candida* yeast infections are the most common complication of DD, occurring in more than 80 % of cases. It has been demonstrated that these infections can act in synergy with bacteria such as *Escherichia coli* to increase the cellular adhesion of the yeasts [3, 26]. Most reports point out the main infectious agent is *C. albicans* (80–90 %). [4, 22, 27]. Other species have been found in lesser proportions: *Candida tropicalis* [28], *Candida parapsilosis* [17] and *Candida glabrata* [27]. It is important to take this into account, because some of these species do not have the same sensitivity to the various antimycotics. *C. glabrata* is especially significant since it does not form pseudohyphae or hyphae, meaning its infection is only regulated by an increase in the number of blastoconidia, and it is more resistant to antimycotics, especially fluconazole.

With the development of new disposable diapers, the incidence and severity of DD have greatly decreased. New technology has allowed superabsorbent polymers such as sodium polyacrylate to be incorporated into the diaper core. These polymers form a gel when they come into contact with urine, reducing skin dampness and friction and helping to normalize skin pH. They can absorb 50–80 times their weight in fluid [11, 16]. The second type of diapers is called breathable diapers [25]. These are made of microporous membranes that enable evaporation while preventing leaks. This reduces the occlusion caused by standard diapers. In some studies, children wearing these diapers had 50 % fewer episodes of DD [16, 19]. Akin et al. [25] conducted a study to assess *Candida* infections in breathable and no breathable diapers. Adult volunteers were inoculated with *C. albicans* at a concentration of 10^6^–10^7^ colony-forming units (CFUs), and yeast survival was demonstrated to be 62 % lower in the breathable diapers. This suggested the “breathing” mechanism has a direct effect on the presence of yeast.

**Fig. 1:**
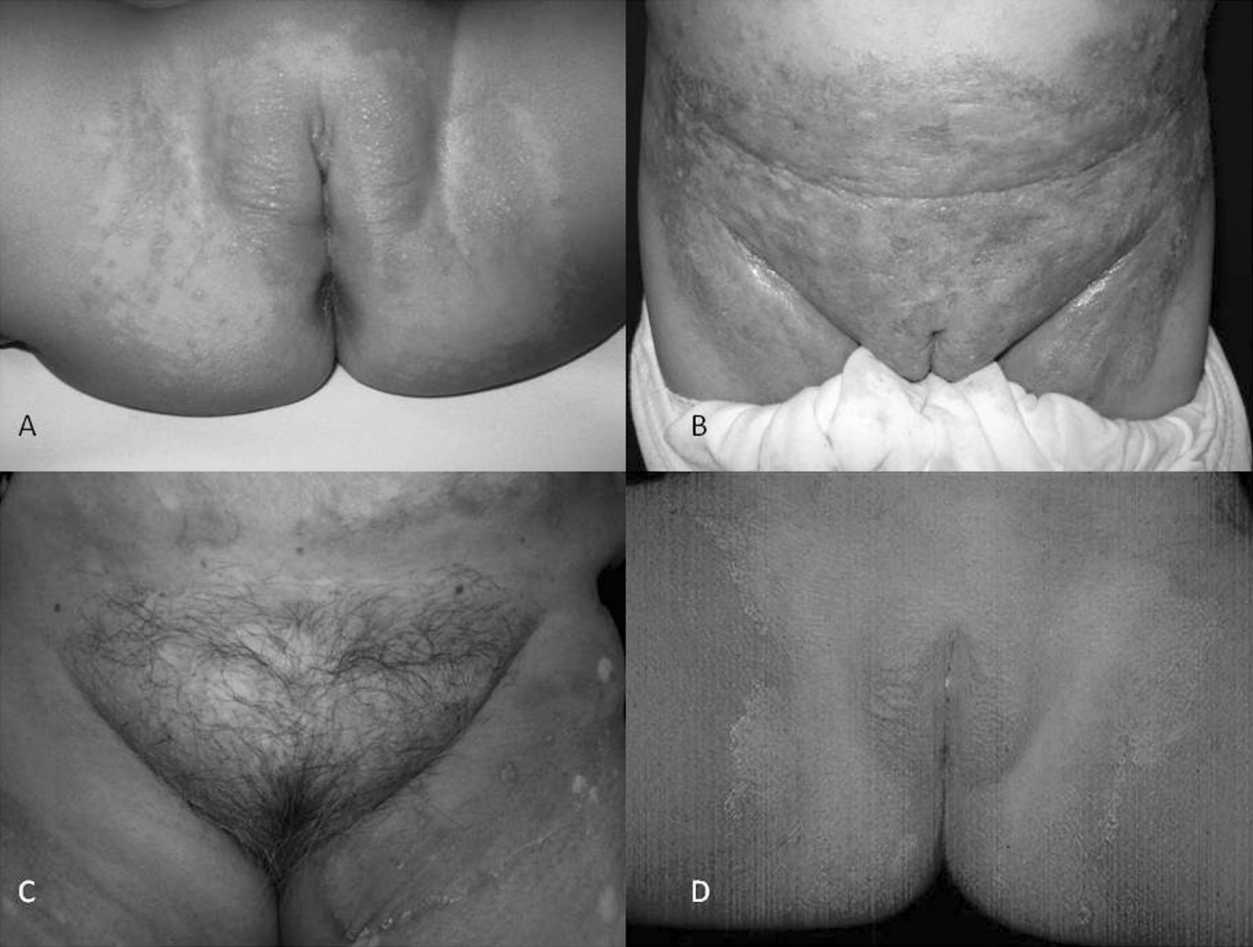
Candidiasis associated with diaper dermatitis. **a** In an infant. **b** In an elderly female patient. **c** Candidosis + dermatophytosis in the diaper area. **d** Tinea in diaper zone

Clinically, dermatitis appears in the region covered by the diaper, affecting the gluteal, perineal and inguinal areas, and occasionally part of the genitals. In more severe cases, it can spread to other regions [1–3, 8, 9]. Early irritant dermatitis is characterized by erythema, mild maceration and edema, while *Candida* diaper dermatitis (CDD) is characterized by erythematous and scaly plaques with maceration and edema, sometimes with satellite pustules or papules, the latter being the most characteristic feature of *Candida* infection. Erosion and ulceration can occur in severe cases. Their symptoms are burning and itching, but these are hard to assess since the condition affects small children [1–3, 7, 9, 11] (Fig. 1).

**Fig. 2:**
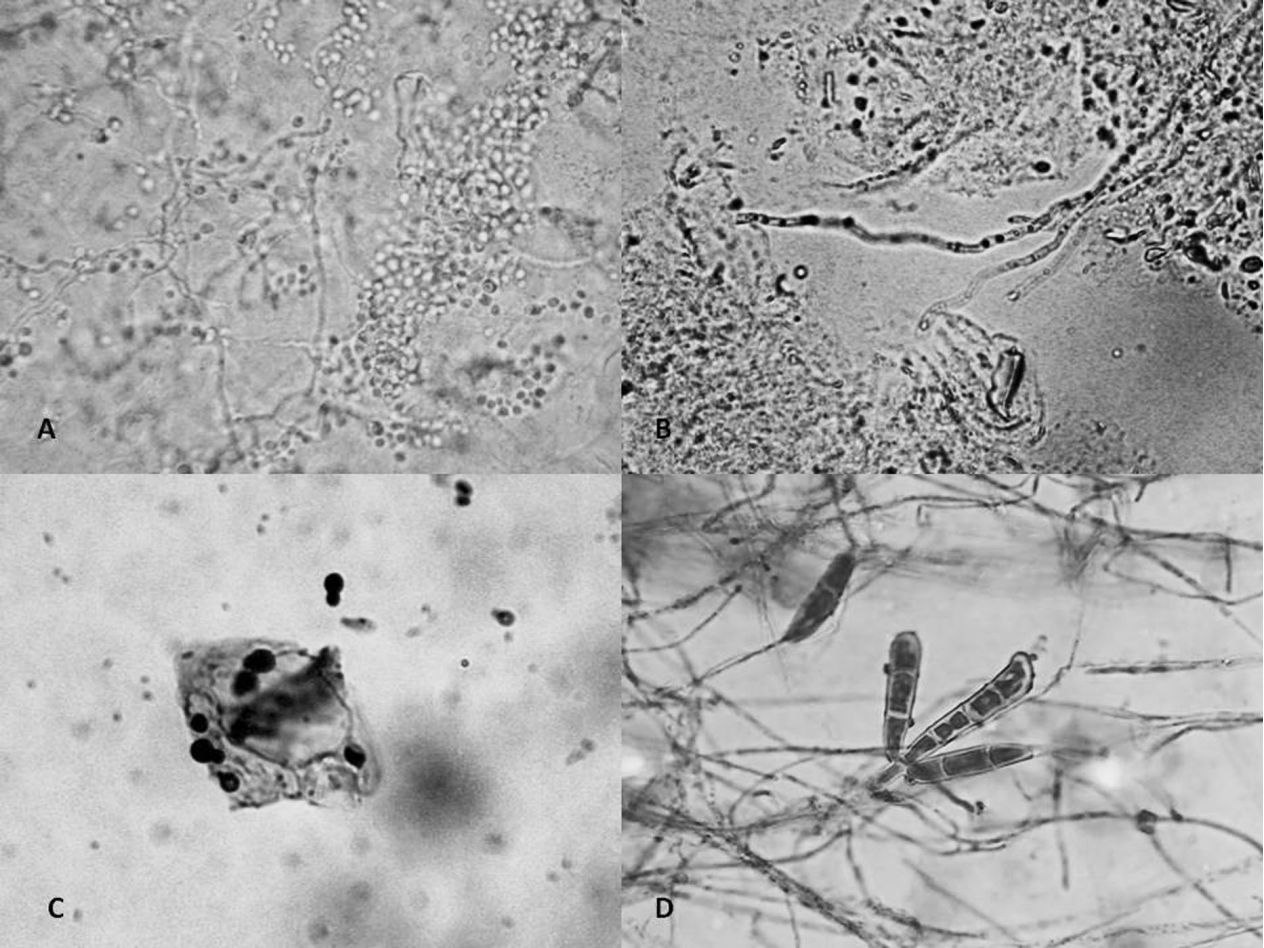
**a** Candidiasis, pseudohyphae and blastoconidia (KOH 10 %, ×40). **b** Dermatophytoses, hyphae (KOH 10 %, ×40). *Malassezia* sp., blastoconidia (Gram. ×100). *Epideromphyton flocossum:* macroaleurioconidia (Cotton blue, ×40)

Disposable diapers are also used in elderly patients who are bedridden or who suffer from any of various conditions: urinary incontinence, mental disorders (Alzheimer’s disease), etc. [27–29]. The clinical characteristics of CDD in the elderly are similar to those found in infants and most patients complain of burning and itching. Foureur et al. [28] conducted a prospective study to evaluate the etiology of DD in bedridden elderly patients. They enrolled 46 patients with an average age of 85 years. The most common cause was candidiasis (by *C. albicans*), which affected 63 % of patients, followed by irritant dermatitis (16 %), eczema and psoriasis (11 % each). This study highlighted the high prevalence of candidiasis and the need for prophylactic use of topical antimycotics [28] (Fig. 2).

Differential diagnoses of CDD include: contact dermatitis, inverse psoriasis, seborrheic dermatitis, atopic dermatitis, dermatophytosis, acrodermatitis enteropathica, impetigo, Langerhans Cell Histiocytosis (formerly: Letterer–Siwe disease), and congenital syphilis [1, 3, 11, 19, 30].

### Laboratory Diagnosis

The diagnosis of DD should be clinical, but mycological cultures are required to confirm CDD. Direct examination with KOH (10 %) should reveal pseudohyphae and blastoconidia, pointing to *Candida* sp. infection; generous collections of blastoconidia are only found in *C. glabrata* infection. When there is important maceration, it is possible to take samples for staining (Giemsa, PAS, Wright) [3, 6, 11, 31]. Samples are normally taken with swabs and can be placed in Sabouraud dextrose agar (SDA), and preferably chromogenic media (CHROMcandida^®^) since the etiological agent can usually be determined with primoisolation. The yeasts obtained can be identified through biochemical testing (zymograms), molecular testing (PCR) and proteomic testing (MALDI-TOF). Biopsies are normally more invasive but are also useful, especially in cases where other diagnoses are possible. Infection is usually seen on the surface (stratum corneum) and in the dermis, with multiple pseudohyphae and blastoconidia [3, 28, 31].

### Treatment

For early DD, the most important thing is to reduce occlusive exposure to urine and feces. This is why frequent diaper changes are recommended. Barrier creams or simple emollients should be used with each diaper change; the best ingredients are zinc oxide, petrolatum, cod liver oil and lanolin [1–3, 6, 7, 11, 19, 30, 31]. The first two ingredients are the most often used. It is important to realize that if there is a *Candida* infection, they are normally counterproductive [19].

Corticosteroids can be used, but only for a short time, i.e., 1–2 weeks at most. They should only be used to treat moderate to severe DD, with intense erythema and irritation, and preferably only if the DD has not responded to inert products [2, 11, 19, 20, 31]. It is better to use low-strength steroids such as hydrocortisone. Although there are medium-to-high-strength steroids on the market with higher anti-inflammatory activity, these are not recommended, as they can cause serious side effects like tachyphylaxis, skin atrophy, striae, granuloma gluteale infantum; also may be systematically absorbed, causing Cushing’s syndrome or hypothalamus–pituitary–adrenal axis suppression [9, 19, 32]. Many of the symptoms associated with steroids may be made much worse by candidiasis [9, 19].

Antimycotics: Since most cases are caused by *C. albicans*, they usually respond well to the various topical antimycotics available. Nystatin is still commonly used [1–3, 11, 19, 31, 33], but is less active than imidazole derivatives. Therefore, if the infection does not respond in 1–3 days of nystatin treatment, switching to azoles is recommended. The most widely used azoles are clotrimazole [1, 3, 7 11, 19] miconazole [1, 3, 6, 11, 19, 33], ketoconazole [11, 19], and bifonazole [28]. They are normally applied twice a day for 7–10 days and generally have high cure rates, ranging from 50 to 68 %.

In an in vitro evaluation study [34] (CLSI M44A method) of strains of *Candida* isolated from candidiasis and CDD, 149 strains were obtained and divided into two groups: *C. albicans* (64.4 %) and *Candida* non-*albicans* (35.6 %). In short, regardless of the species of *Candida*, clotrimazole had around 20 % resistance and ketoconazole had around 35 % resistance, while nystatin was 100 % sensitive to the *C. albicans* strains and only showed 11 % resistance to non-albicans strains. These latter data are interesting since they show that although the old treatments are effective, they can display resistance of 10–35 % [34].

Ciclopirox is one of two new antimycotics that have been used to treat CDD. Galllup et al. [35] conducted an open-label, non-comparative study using a ciclopirox suspension (0.77 %) applied twice daily for 7 days and assessed after 14 days. They obtained good results in total success scores (*p* < 0.047), as significantly decreased signs and symptoms and mycological cure were found on evaluation.

Sertaconazole nitrate is a broad-spectrum third-generation [36]. It is a fungicide with long skin perdurability (up to 72 h), which is one of the properties that makes it different from other imidazoles. Bonifaz et al. [37] conducted a study on CDD and obtained the following results: Sertaconazole cream (2 %) was applied twice daily for 14 days, causing a decrease of more than 50 % in clinical signs after 7 days of treatment, with 88.8 % clinical and mycological cure. It was particularly effective against non-*albicans* species and caused only one side effect (3.7 %), namely dermal irritation. We consider this drug to be a new alternative to treat CDD since it has a good efficacy–safety ratio [37].

The use of oral antimycotics is only restricted in severe cases and cases associated with other types of candidiasis (oral and gastrointestinal). Nystatin suspension has been administered at a dose of 1 ml 3–4 times daily (100,000 IU), as has fluconazole at a dose of 3–6 mg/kg/day [11, 19, 31].

## Dermatophytosis in the Diaper Area

### Epidemiological, Pathogenic and Clinical Forms

Dermatophytosis or tinea infections can affect the diaper area. This is called dermatophyte diaper dermatitis and has been studied for many years. It occurs much less often than CDD [4, 38–42]. The most frequently isolated etiological agent is *Epidermophyton floccosum*, found in around 80 % of isolations, followed by *Trichophyton rubrum*. There are also some isolated cases of *Trichophyton interdigitale* (*T. mentagrophytes* var. *interdigitale*) and *Trichophyton verrucosum* [38–42].

The most reports are from the 1980s. Though few cases are published nowadays, they undoubtedly still occur [38–42]. This condition develops differently from CDD since dermatophytes, unlike yeasts, are not found among normal flora; external infection is required. Most cases reported had some relation to the parents or caregivers of the children. Almost all patients had tinea pedis (most often by *E. floccosum*) [38, 40, 42], and some had onychomycosis (*T. rubrum*) [4, 39, 42], which suggests the dermatophyte spores were transmitted from hands or fomites and benefitted from the moist conditions and high CO_2_ concentrations, which are known to stimulate dermatophyte growth [24, 25].

Dermatophytosis has the following clinical characteristics: it appears in the whole area covered by the diaper, affecting the lower abdomen, gluteal and inguinal regions, and upper third of the thighs, and can spread to the waist. The genitals are not affected unless the condition is associated with steroids that are high in strength and/or used over a long period of time. Morphologically, the condition presents erythematous, scaly dry plaques with active vesicles and borders and no satellite lesions, with satellite papules only occurring in exceptional cases, when there is a mixed infection (dermatophyte + *Candida* sp.) (Fig. 1). The condition is related to corticosteroid treatment [38–42]. The most common symptom is itching. There have been few comparable reports in the literature on elderly patients who use disposable diapers, but there are more cases since adults more often have tinea pedis and onychomycosis. This infection is likely to go unnoticed [41, 42]. The differential diagnosis includes CDD, inverse psoriasis, atopic dermatitis, congenital syphilis and seborrheic dermatitis [4, 38, 42].

### Laboratory Diagnosis

The simplest test consists of taking scrapings from the scaly areas and performing direct examinations with KOH (10 %), which should reveal long, thin hyphae. Dermatophytes develop slowly in SDA and SDA + antibiotics, and are identified based on macro- and micromorphological characteristics [41, 42].

### Treatment

If the condition is associated with DD, the same treatment measures should be taken and a topical antimycotic must be added. The most widely used antimycotics are clotrimazole, miconazole and ketoconazole [42, 43]. Ciclopirox [35, 43] and sertaconazole [36] are also effective. It is important to note that nystatin and mupirocin are not effective against dermatophytosis [19]. Treatment time should be a little longer, 2–3 weeks with one or two applications, depending on the antimycotic selected. It is important to locate the source of the infection and treat it to avoid reinfection.

## *Malassezia* sp. in the Diaper Area

Some species of *Malassezia* can contribute as exogenous flora to develop various diseases. They have been found in seborrheic dermatitis, atopic dermatitis and psoriasis. They are not believed to be etiological agents [44–49], but do contribute to exacerbation of the condition or as allergens. They can produce specific antibodies and increase the responses of immune cells and IgE, i.e. *Malassezia* spp.-IgE. This antibody in particular is usually a severity marker in atopic dermatitis. It has been confirmed that an increased number of *Malassezia* yeasts activates the alternative complement pathway, leading to an inflammatory process and therefore erythema and flaking. It has also been demonstrated that *Malassezia* yeasts produce enzymes such as phosphatases and lipases, which contribute to activation of the process [44–49].

It should be noted the three conditions mentioned can also occur in the diaper area. Seborrheic dermatitis occurs most often on the scalp, but in children it can spread to the torso and diaper area. Atopic dermatitis can affect nearly all skin and is aggravated in the diaper area. The diaper area is the most frequent location of psoriasis, especially inverted psoriasis. The number of yeasts present is variable, but lower than on the skin of the scalp [45, 47]. The most commonly found species are *Malassezia restricta*, *Malassezia sympodialis*, *Malassezia dermatis* and *Malassezia globosa* [44, 45, 47, 49].

They are easily recognized with stains such as Gram or Giemsa, which reveal a variable number of yeasts. They are isolated on special media such as SDA + olive oil or in modified Dixon’s agar. They are identified using biochemical methods (assimilation of surfactants) or molecular methods (PCR) [47].

In cases in which yeast numbers increase, another course of treatment must be added. The most responsive topical antimycotics are ketoconazole, ciclopirox and sertaconazole. The first two are available as shampoos, and their use is suggested to help clean the diaper area [49, 50]. Finally, it is important to highlight erythrasma is an infection so similar to diaper candidiasis and dermatophytosis, and this is a pseudomycosis due to *Corynebacterium minutissimum* (Gram-positive, coryneform actinomycetal). This disease occurs in skin folds as erythematous, scaly, well-defined plaques, covered by a fine; most cases are asymptomatic or cause slight pruritus and clinically indistinguishable from candidiasis and intertriginous tineas [6, 50–52]. Laboratory diagnosis is made with Wood’s light, the plaques fluoresce a coral-red color, and should be confirmed with Gram-positive filaments and cultures in blood agar or brain–heart infusion (BHI) broth and identified through biochemical or molecular testing [6, 51, 52]. The treatment of choice is topical erythromycin, applied once or twice a day for 1 week. Fusidic acid and mupirocin can also be used. Some antimycotics are active against these bacteria; the most important bifonazole, sertaconazole and ciclopirox. Pharmacodynamics could explain many undiagnosed cases resolved with their use [7].
